# Supplementary material for: Erlotinib Promotes Ligand-Induced EGFR Degradation in 3D but Not 2D Cultures of Pancreatic Ductal Adenocarcinoma Cells
Source: Cancers (Basel). 2021 Sep 7;13(18):4504. doi: 10.3390/cancers13184504 (PMC8470972; doi:10.3390/cancers13184504)
Supplement: Supplementary file 1 [file cancers-13-04504-s001.zip › cancers-1334716-supplementary.pdf]

Supplementary Materials

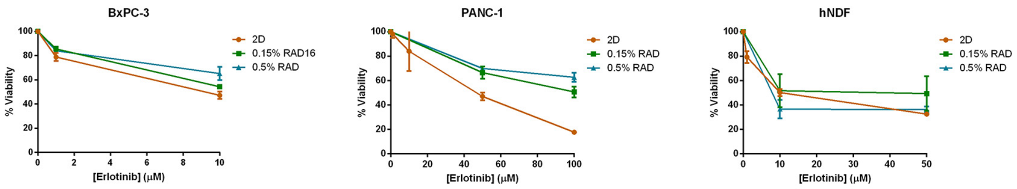

Figure S1. Erlotinib dose-response curves in 2D and 3D cultures (0.15% and 0.5% RAD16-I) of BxPC-3, PANC-1 and hNDF cells.

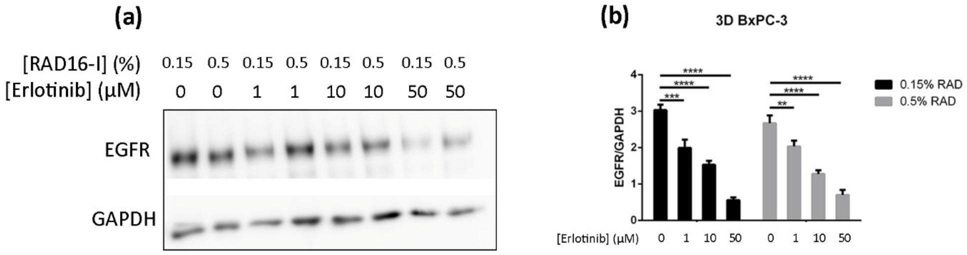

Figure S2. EGFR degradation with increasing concentrations of erlotinib in EGF-incubated BxPC-3 cells in 3D RAD16-I scaffolds. (**a**) Western blot bands of EGFR in BxPC-3 cultured in soft and stiff RAD16-I scaffold with different erlotinib concentrations; (**b**) Densitometry of bands showed in (**a**). GAPDH was used as an internal control. Statistical differences are indicated as ** for pv<0.01, *** for pv<0.001 and **** for pv<0.0001.

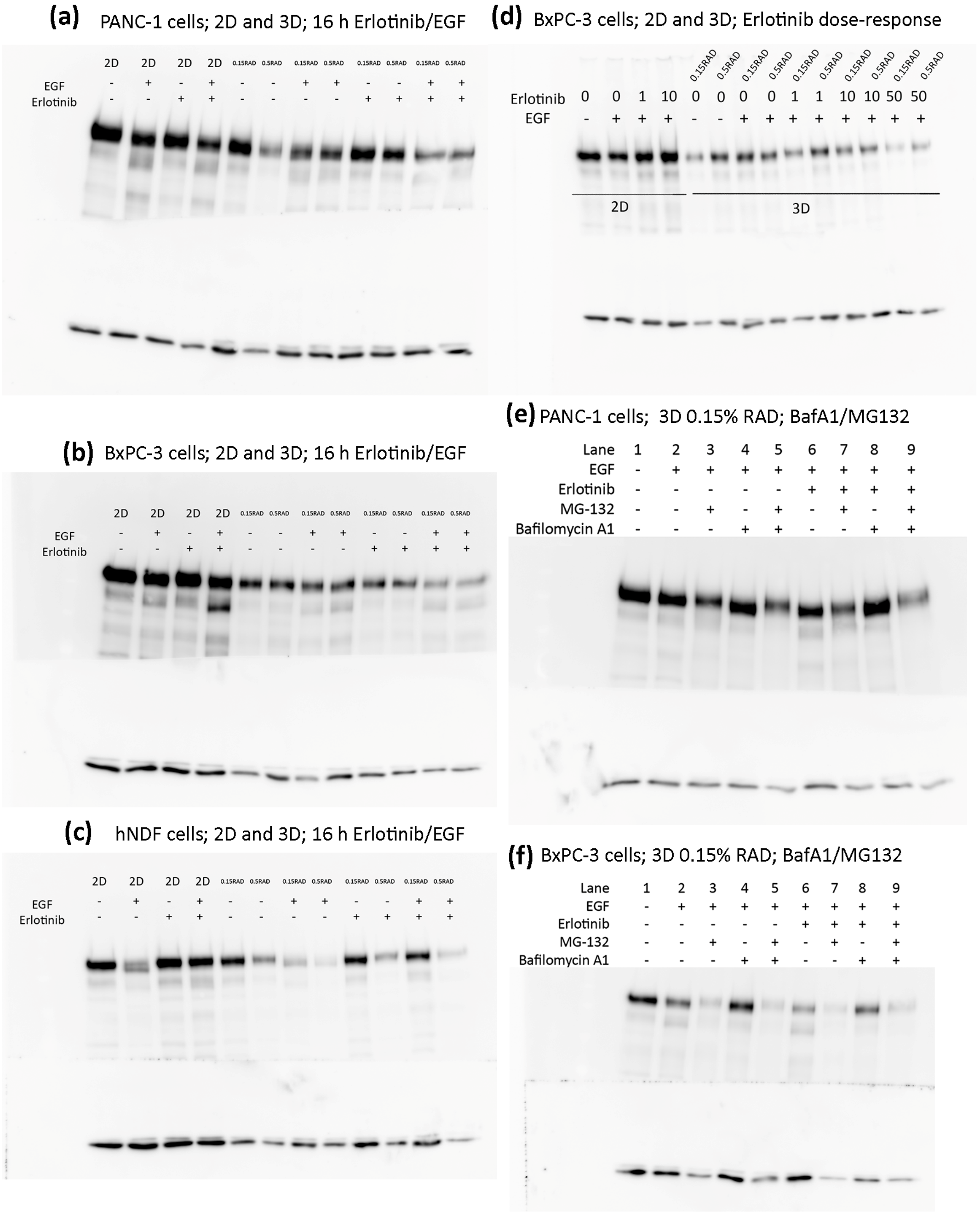

Figure S3. Uncropped western blot images. Blots from Figure 7 (a,b,c), Figure S2 (d) and Figure 8 (e,f).
